# Barriers and enablers for sufficient moderate-to-vigorous physical activity: The perspective of adolescents

**DOI:** 10.1371/journal.pone.0296736

**Published:** 2024-02-16

**Authors:** Viktoryia Karchynskaya, Jaroslava Kopcakova, Andrea Madarasova Geckova, Boris Katrusin, Sijmen A. Reijneveld, Andrea F. de Winter

**Affiliations:** 1 Department of Health Psychology and Research Methodology, Faculty of Medicine, P. J. Safarik University in Kosice, Kosice, Slovakia; 2 Department of Community & Occupational Medicine, University Medical Center Groningen, University of Groningen, Groningen, The Netherlands; 3 Olomouc University Social Health Institute, Palacky University Olomouc, Olomouc, Czech Republic; 4 Institute of Applied Psychology, Faculty of Social and Economic Sciences, Comenius University in Bratislava, Bratislava, Slovakia; 5 Department of Psychological Sciences, Constantine the Philosopher University in Nitra, Nitra, Slovakia; Fred Hutchinson Cancer Research Center, UNITED STATES

## Abstract

**Background:**

Interventions to improve physical activity (PA) among adolescents continue to be a public health priority. To promote PA more effectively, we need to identify the main factors contributing to (not) engagement in PA in the perspective of adolescents themselves. Thus, we explored the barriers and enablers for sufficient moderate-to-vigorous physical activity (MVPA) in adolescents from their point of view.

**Methods:**

We used qualitative data collected as part of the international Health Behaviour in School-Aged Children study. We obtained data from 14–17 years old adolescents from the first year of Slovak high school. We conducted 11 online, semi-structured individual and group interviews with 24 participants in total (7 boys; mean age = 15.17, SD = 0.87) in Slovakia. We analysed the data using consensual qualitative research and thematic analysis.

**Results:**

In the statements of adolescents, four main themes were identified regarding factors contributing to (not) engagement in PA among adolescents. ‘Myself as a source’ represents the importance of adolescents’ own efforts, knowledge, physical predispositions and PA experience. ‘How PA can be done’ represents school as an opportunity for PA, and PA teachers and sports coaches as specialists who can create an enabling environment for sports. ‘Others as a source’ represents the social circle that can set a positive sports example and can encourage adolescent’s efforts in PA. ‘Factors outside’ represents other factors that can inspire adolescents, e.g. by giving them a comfortable space and time to exercise, or can be a barrier to PA.

**Conclusion:**

The potential factors that include adolescents’ perspectives can be more leveraged in designing supportive, inclusive, enjoyable, and skills-appropriate PA programmes.

## Introduction

Physical activity (PA) has a positive impact on the physical and mental health of youth [1, 2], but despite the number of physically inactive adolescents is increasing every year. According to Inchley et al. [3], only 19% of adolescents achieve the recommended 60 minutes of moderate-to-vigorous physical activity (MVPA) daily. Moreover, the lack of PA leads to such diseases as obesity, diabetes, and cardiovascular diseases [4]. As a result, physical inactivity has become the fourth leading determinant of global mortality [5]. This demonstrates that the interventions for the improving PA among adolescents continue to be a public health priority.

In order to develop effective intervention programs, it is necessary to better understand the reasons why adolescents are (not) engaged in PA [6], i.e. which barriers and enablers play a role. As PA is influenced by many different factors, also Bronfenbrenner’s ecological systems theory [7] has been suggested that participation in PA is determined by factors at the intrapersonal, interpersonal, organisational/environmental, and policy and legislative levels [8]. Evidence from quantitative studies have shown that factors strongly influencing adolescent engagement in PA are directly related to the adolescent himself [9–11]. These include psychological factors (e.g. goal orientation, attitude, self-efficacy, behavioral control) and biological factors (e.g. physical predisposition, physical abilities). As noted by Abdelghaffar et al. [12], age, gender, and socioeconomic status also have a significant impact on how physically active adolescents are. In addition, the social and the physical environment can have a constant impact on adolescent engagement in PA, in supporting adolescents’ PA [13], and in providing access to opportunities for PA [14], respectively. However, a disadvantage of such quantitative surveys is that they usually address a narrow set of predetermined factors, which are prioritized by adults [15].

The above-mentioned limitations of the available quantitative evidence might be overcome at least partially by a qualitative approach to better understand the factors influencing PA by exploring the point of view of adolescents themselves. Previous qualitative studies have already shown that the main barriers that prevent adolescents from being physically active include: negative PA experiences at school, self-doubt about appearance, restrictions from family and friends, lack of green parks and sports fields, and lack of opportunity to be active at school [16–19]. In addition, adolescents identified as PA enablers: positive experiences with PA, personal factors (e.g., fun, perceived competence), support from family and friends, and access to physical education programs [19–23]. However, most of these studies have been carried out in Australia, Canada, the US and the UK, while other regions have been covered hardly. In order to create programs that help to increase and maintain the level of physical activity among adolescents effectively, we should identify the main barriers and enablers for physical activity, from the perspectives of adolescents themselves. That information currently lacks. Thus, more evidence is needed on the adolescents’ perception of barriers and enablers for PA in other regions and groups as environmental and contextual differences are likely, e.g. due to differences in policies and culture. In sum, our goal is to enrich existing findings by exploring perspectives of adolescents and particularly adolescents from Central and Eastern Europe. Therefore, the aim of this study was to explore the barriers and enablers for sufficient MVPA in adolescents from the perspectives of adolescents.

## Methods

### Design of the study

To obtain an in-depth insight into adolescent’s perceptions of barriers and enablers of PA, a qualitative research design was used, embedded in the Health Behaviour in School-aged children (HBSC) study, consisting of individual and group interviews provided in selected high schools in Slovakia. The individual and group interviews were selected for this study as the most suitable technique as they have been proven to be an effective method in gathering qualitative data among adolescents. These interviews help to create interactive conversation, evoke memories, help participants to verbalize their responses and enable testing the consistency of statements [15]. In addition, we used a consensual qualitative research (CQR) methods and thematic analysis. As noted Hill et al. [24], CQR methodology focus on the different experiences and opinions of the research team members and the conclusions they are able to draw from their assumptions.

This qualitative study was conducted in accordance with the consolidated criteria for reporting qualitative research (COREQ) [25] and in accordance with the ethical standards outlined in the Declaration of General Assembly of the World Medical Association [26]. The study protocol was approved by the Ethics Committee of the Medical Faculty (19N/2020) at the P. J. Safarik University in Kosice.

### Study setting, sampling and participants

We obtained data from 14–17 years old adolescents from the first year of Slovak high school. We conducted 11 individual and group interviews with 24 participants in total (7 boys; mean age = 15.17, SD = 0.87). The participants were selected from all types of high schools in Kosice region, Slovakia; i.e. grammar schools (with A levels graduation), secondary schools with GCSE graduation and secondary schools with apprenticeship certificate graduation.

We performed the sampling for this qualitative study in several steps. Firstly, we contacted the school administrators to inform them about our study. After obtaining their consent for participation in the study, we contacted the parents of the potential participants and obtained their informed consent via an online platform. If the parents agreed with their child’s involvement, we got in touch with the adolescents and obtained informed consent from them as well. Communication with parents and adolescents was provided by class teachers via the online platform EduPage (EduPage is a cloud based school management system which enables communication between school/teachers/school administrative and adolescents/parents or care-takers) to avoid that the researchers obtained contact information. Participation in the study was fully voluntary and confidential, and all respondents were allowed to withdraw at any time.

### Procedure and measures

We conducted online semi-structured group interviews in the period between November 2020 and June 2021, by Zoom because of COVID-19 regulations. Prior to the interviews, adolescents were asked to fill out a questionnaire that included information about gender, age, location (city, village), type of school (secondary school; secondary vocational school, i.e. with general certificate of secondary education; secondary vocational school, i.e. with vocational certificate), frequency of PA, and participation in organized leisure activities. We measured adolescents’ PA by asking adolescents about the number of days in the past week that they were physically active for a total of at least 60 minutes per day, and if they did any team or individual sport in their leisure time, with response categories “yes” and “no” [27]. We then categorised adolescents’ PA in: (1) inactive: adolescents who were active as defined above less than 5 days per week and were neither engaged in team, nor in individual organised sports, (2) active: active 5–7 days per week or engaged in team or individual organised sports, and (3) very active: active 5–7 days per week and also engaged in team or individual organised sports [28]. Quantitative descriptive characteristics of the sample are presented in Table 1.

**Table 1:** Characteristics of the sample (N = 24, 14-17-year-old Slovak adolescents, data collected in 2021).

Characteristic	N (%)
**Gender**	
Boys	7 (29.2)
Girls	17 (70.8)
**Age**	
14 years old	5 (20.8)
15 years old	12 (50.0)
16 years old	5 (20.8)
17 years old	2 (8.3)
**Location**	
City	15 (62.5)
Town	2 (8.3)
Countryside	7 (29.2)
**Type of school**	
Secondary school	12 (50.0)
Secondary vocational school (with GCSE)	10 (41.7)
Secondary vocational school (with vocational certificate)	2 (8.3)
**Adolescents‘ PA**	
Inactive	9 (37.5)
Active	11 (45.8)
Very active	4 (16.7)

Notes: PA–physical activity; GCSE–general certificate of secondary education; Inactive–less than 5 days moderate-to-vigorous physical activity and no engaged in team or individual organised sports; Active– 5–7 days moderate-to-vigorous physical activity or engaged in team or individual organised sports; Very active– 5–7 days moderate-to-vigorous physical activity and engaged in team or individual organised sports.

We then conducted 11 individual and group semi-structured interviews. The interviews were conducted by a trained psychologist (BK) with extensive work experience with adolescents on an online counseling platform. The members of the research team (AMG, JK, VK) were professionals with various backgrounds in psychology, and they all were a part of the interviews as silent observers.

The interviews were conducted using a thematic guide that included the following questions:

1. Although many adolescents say that they are physically active every day, we find that many young people do not have sufficient physical condition and have overweight problems. How is it possible? What is the reason for it?
2. What is it that makes some adolescents more active, some less? (What causes it?)
3. What could you or adults do / change to be more physically active? (To make you want to move, to make it attractive, interesting, "hot" and so on…)

The interviews lasted for approximately 45–60 minutes and were audio- and video-recorded. The interviews were conducted in the Slovak language and done via the online platform Zoom, as they were conducted during the second wave of Covid-19 pandemic in Slovakia and due to government measures Slovaks did not have a chance to meet with participants face-to-face. In addition, the records of the online discussions were rewritten for further analysis while maintaining the anonymity of the respondents. Those parts that could potentially lead to the identification of participants was omitted.

### Data handling and analyses

Regarding data handling, we processed the obtained data by transcribing audio- and video-recorded interviews verbatim in Slovak. Next, the transcriptions were checked to ensure their accuracy and then uploaded into MAXQDA standard platform (VERBI Software, Berlin, Germany) for data analysis. We coded the data using the CQR methodology and thematic analysis. The team of coders consisted of a lead investigator (AMG), a senior (JK), and junior researchers (VK, BK), all of whom were trained in the CQR methodology. First, members of the research team carefully read transcripts and field notes, segmented texts and created codes. Generation of codes was realized individually and after the completion of the coding process, members exchanged coding outputs for cross-checking of coding quality. In case of disagreements or re-coding appeals, members initiated coding meetings and discussed problematic codes until the consent in the final output of coding was achieved.

In the analysis, we first described our study sample using the data from the questionnaires. Next, members of the research team went through the entire transcript using thematic analysis and identified the barriers and enablers for sufficient MVPA as perceived by adolescents. The codes produced during data handling were clustered into subthemes and themes. All team members first did this individually. They then met to share the created subthemes and themes and discussed until a consensus was reached on the final thematic model of the barriers and enablers for sufficient MVPA in adolescents.

## Results

### Background characteristics

Of our respondents, 62.5% were from cities, 50.0% went to secondary school and 45.8% were physically active. Half of the respondents were aged 15 and 70.8% were girls (Table 1).

### Main themes

**Fig 1:**
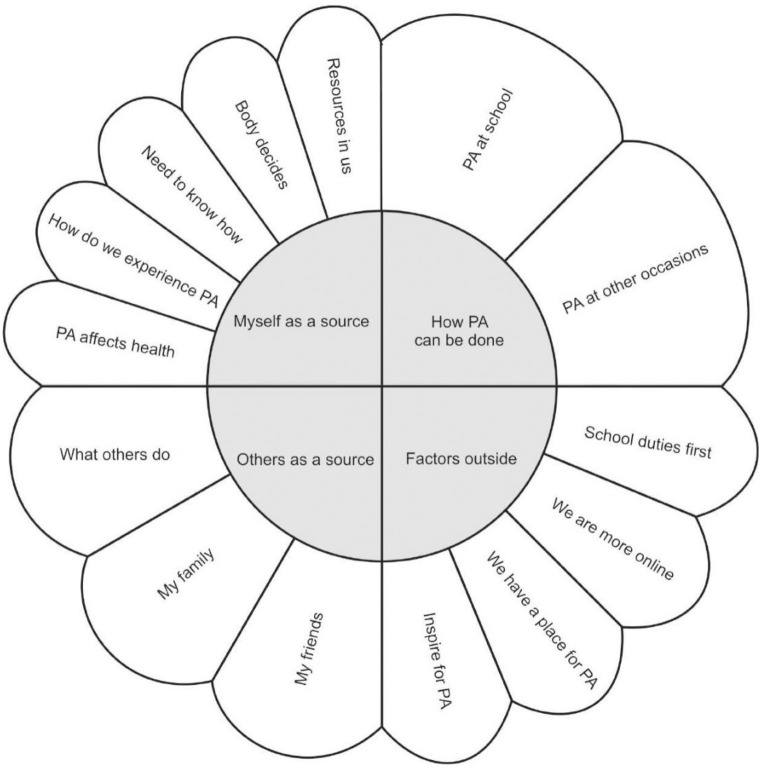
Factors contributing to (not) engagement in PA among adolescents. Note: PA—physical activity.

We identified four main themes regarding factors contributing to (not) engagement in PA among adolescents: 1. ‘*Myself as a source’*, 2. *‘How PA can be done’*, 3. *‘Others as a source’*, 4. *‘Factors outside’*. The themes are shown in Fig 1 and will be described in more detail below in Table 2, in S1 Table (in English language) and in S2 Table (in Slovak language).

**Table 2:** Quotes illustrating factors contributing to (not) engagement in PA among adolescents.

Themes/*Subthemes*	Quotes
**Myself as a source/ *Health benefits***	
The positive effect of sports on physical health motivates	*“… I started training about a month and a half ago*, *and I have to say that I see results in terms of fitness*. *I just actually see that I can handle it more and more… I do not know*, *but I feel good after training… I am more tired and even better to go to bed sooner…”*
The positive effect of sports on mental health motivates	*“… but it also has a positive effect on me in terms of my mentality or something… I have to say those pressures and everything are somewhat distracting*, *when there is stress from school*. *I do not think about it when I exercise*, *for example… in my opinion*, *I would recommend it to those who are prone to those depressions and so that something to distract*.*”*
**Myself as a source/ *How do we experience PA***	
Success motivates	*“So*, *what I think motivates people to play sports is definitely the success*. *Because when people see that they are doing well*, *they just go up and try to reach their greatest peak…”*
Failures demotivate	*“… that many times*, *when someone was not good in PA since childhood and I was exactly like that… When I came to school*, *I was clumsy in football*. *You cannot even look on it*. *Once*, *I pushed the ball and almost broke the window*. *So not many people chose me for the team…”*
**Myself as a source/ *Need to know how***	
They do not know how much and how to exercise	*“*… *for example*, *someone does an activity*, *they need to know how to do it properly*. *Even when you exercise*, *it may hurt you*.…*I do not know for sure*, *but maybe the training was not as effective as it could be*.*”*
To be able to try different physical activities	*“*… *in order to find some sports that you will really enjoy*, *you will need a help to show you what those sports are like; or let you look more and find it by yourself*, *what you like best and then you would actually go into the fact that ‘I like this*, *I will play this‘*, *or just like that*.*”*
**Myself as a source/ *Body decides***	
Physical predisposition, physical skills and fitness help	*“Well*, *I think it could also be that someone is built for sports*. *Because*, *for example*, *my friend is not just a person who was built for sports*. *He does not do very well on the physical education*, *or anything like that*, *so when he is free*, *he does not just go out and play sports*…*”*
Health problems as a PA barrier	*“*… *medical condition*, *so my classmate actually had to finish ballet because she has such an illness as asthma and her trainer said she had to quit*. *The doctor told her the same and she is already quite demotivated*. *That she just misses ballet*, *the dancing*.*”*
**Myself as a source/ *Resources in us***	
Laziness	*“I think laziness plays a big role in that*, because I have a lot of people like peers who are extremely lazy and just cannot get into things like that (*PA). *They will say*, *‘Well*, *I had rather play on PlayStation some video games’*.*”*
To show determination	*“So*, *it is basically about trying to get over ourselves and trying to do things that do not always suit us*. *Do not go into your comfort zone*, *but go out of it by doing things differently*.*”*
**How PA can be done/ *PA at school***	
Physical education in schools as a duty and boredom, not joy	*“… or still do it so that the exercise is not actually a punishment or an enemy*, *but that we actually take it gladly*.*”*
Teacher as a facilitator	*“Yeah*, *well*, *our gym teacher told us*, *‘I live in Myslava [place near Kosice]*. *I go jogging every morning*. *You can join whoever wants‘*.*”*
**How PA can be done / *PA at other occasions***	
Personality, experience and attitude of the coach	*“Or even if we are already at that training*, *you can just sit down with your coach once in a while and ask him ‘What I should improve*?*‘ or*, *What I cannot do*?*‘*, *and then he can also tell us simply what is good and what is bad for us*.*”*
When PA is fun	*“I perceive the sport as fun*. *I enjoy it*. *I can go out with friends*…*and of course*, *I feel better when I exercise*.*”*
**Others as a source/ *My friends***	
I am going where my friends are	*“So*, *I think it can also be the team*, *because*, *for example*, *I personally just do not go cycling alone*, *because I do not like it*, *but if I am going with someone*, *I am out for 3–4 hours*…*”*
The influence of the collective	*“So first of all*, *it is a team*, *one hundred percent true*. *Because when I found good friends in the team*, *I really do not want to leave this team*…*”*
**Others as a source/ *My family***	
If someone does not play sports as a child, it will be difficult later	*“As I think*, *when person did not play sports as a child*, *so when he is older*, *it is getting harder for him*, *as when he did sports since childhood*…*”*
My family supports me in PA	*“Or maybe family and neighbourhood support*. *Because when they do not support person*, *he does it unnecessarily*. *That no one believes in him and he is just alone in it*…*”*
**Others as a source/ *What others do*?**	
The new generation is more passive and more striving for comfort	*“*…*overall*, *young people move less*, *because it does not matter if we are at school or at home*, *we are still sitting*. *We are sitting on a chair either behind the desks or at the computer*. *Some may have around 14 hours per day and they spend most of that time either on a chair or when they are going by bus or whatever*, so that their body may not be used to it (*PA)… *they do not have to deal with it much*.*”*
Among the surroundings there are very few who do PA every day	*“So*, *in my opinion*, *they probably did not give true information*. *Because just from the way I notice my surroundings*, *I probably do not know any young person who would train or have any PA 6–7 times per week*.*”*
**Factors outside/ S*chool duties first***	
To fulfil school duties in the first place	*“*… *Because when we study in person*, *we need to go to school*. *I leave in the morning and come back in the afternoon*, *yeah*, *around 16*:*00*. *So*, *there is really little time space that I can find for some sport activities*. *So I come back home*, *eat something or whatever*, *and I start to do things for school*. *It takes a long time*, *yeah*, *because there is a lot of learning*, *writing*, *tests and whatever*. *So*, *in the evening*, *when I am done all my stuff*, *maybe it could be around 21*:*00*, *but I am really going to say that I do not want to do anything*, *because I am tired*.*”*
Fatigue from school	*“It may still be that person is stressed out*. *So*, *he actually does not do as well as he should and he is stressed out of school*, *for example*. *So*, *he just does not have the motivation to train*…*”*
**Factors outside/ *We are more online***	
Videos, groups and posts on the internet are motivating	*“So*, *if the person is a positive influencer*, *such as Attila Végh [Slovak athlete]*, *which actually presents himself on these social networks as a person*, *not only as an athlete*, *but also presents his life and his family*… *It is not just that he brags about what he won and his achievements*, *and also those athletes who help some sick children*, *so that is actually the positive role model*, *is not it*?*”*
Everyone is online	*“It can be said*, *that when everyone is on the computer*, *this social contact is maintained through the computer*. *So this is how they satisfy this social need through their own "Discord" and other things that they can talk with whole internet community*.*”*
**Factors outside/ *We have a place for PA***	
PA-promoting environment	*“So*, *in my opinion*, our environment is also affects it (*PA). *For example*, if I live far from something (*sport clubs), *I just prefer to watch the series at home myself*…*”*
Available sports clubs	*“Well*, *it may also be that some sports are too expensive and someone cannot attend them*, *or rather the approach to those sports*. *For example*, *if I want to attend a sport*, *I have to go through the whole city because there is simply nothing in my area*. *Or in schools*, *there are just a few such sports clubs*…*”*
**Factors outside/ *Inspire for PA***	
Famous person as a role model	*“… if we have a role model in someone*, *that one day we would like to be like him/her*, *we go for it*, *and when the others do not really have anyone like that*, *nothing motivates them to be better*.*”*
Sports campaigns as a means of PA support	*“In my opinion*, *it is important to make more promotion among the students*, *and support those sports in general*. *Now there is one such project*… *where the sport is actually made in more attractive way with the help of those influencers*, *as Nina [Slovak name] said*.*”*

1. **Myself as a source** included the following subthemes:
  - *‘Health benefits’ regards that* an enabler for PA is the perceived positive impact of sports on mental and physical health. In addition, adolescents pointed to health and appearance as PA enablers.
  - *‘How do we experience PA’* regards that the enabler for PA is a sense of success. Moreover, well-being after training, progress in sports, and support from others were noted as PA enablers. As the main barriers in this subtheme adolescents identified failures and inexperience in sports, which are highly demotivating for them.
  - ‘*Need to know how’* regards that it is necessary to find time and plan sports activities, and it is also important to master the technique of doing exercises, gradually improving your physical shape, and use energetic music. In order to stay physically active, according to adolescents, regularity and perseverance are necessary, you need to find meaning in PA and make it a habit. In addition, it was noted, that you should pay attention to diet and sleep, choose the appropriate load and frequency of PA. Moreover, adolescents pointed out the importance of being able to try different types of sport and find what you like. As a barrier for PA it has been noted that some adolescents do not know how much and how to exercise.
  - ‘*Body decides’* regards the importance of physical predisposition, physical skills and fitness as the enablers for PA. As the main barriers they identified: lack or loss of physical condition, overweight and health problems. In addition, adolescents noted as a barrier the injuries and the fear to be injured during PA.
  - *‘Resources in us’* regards the importance of setting goals for PA, the ability to take responsibility and show determination, as well as the ability to expand a comfort zone and overcome laziness, pain and reluctance to exercise. Moreover, the need to have a connection with sport was highlighted as the enabler. As the main barriers for PA in this subtheme adolescents identified laziness, a quick loss of interest in PA and the presence of other hobbies that are not related to PA (e.g. drawing, music, reading, etc.).
2. **How PA can be done** included the following subthemes:
  - ‘*PA at school’* regards that a major enabler for PA could be an increase of the number of hours of physical education, which is currently not enough at school (i.e. two times per week). The teacher’s enthusiasm and attitude towards PA of students were also mentioned as the enabler. In addition, adolescents indicated that a PA enabler can be that the school offers opportunities to be physically active (e.g. more sport clubs, the ability to play ping-pong or darts through breaks). Adolescents pointed out that school physical education should be more friendly and entertaining, should take into account the physical abilities of students and select the appropriate PA for them, and should give more sports to choose from. Moreover, adolescents highlighted the condition and equipment of sports halls in schools as PA barrier.
  - *‘PA at other occasions* regards the importance of trainers’ personality, attitude towards students and his experience, which can act as an enabler and a barrier for PA. In addition, adolescents point out the need to make PA more fun and attractive, e.g. PA should be carried out in a playful way with more games and new exercises. The high cost of some sports was noted as a barrier.
3. **Others as a source** included the following subthemes:
  - *‘My friends’* regards that the enablers for PA are the support and encouragement from friends, e.g. inviting to play sports together. Adolescents noted that they do not like and are not interested in doing PA on their own; they go where their friends spend time. In addition, as PA enabler was mentioned the importance of building relationships with peers during sports. In team sports, adolescents emphasized the mutual support from team. As PA barrier was noted an unsuccessful experience in team sports, which discourages the desire to play sports.
  - *‘My family’* regards that the enabler for PA is the support from parents and relatives. According to adolescents, the engagement in sports from an early age, as well as the decision of parents to involve the child in PA helps to overcome the initial reluctance to play sports. Moreover, as PA enabler was highlighted the parents role model. As barriers to PA were noted the pressure from parents on the child to play sports as well as the economic situation in the family.
  - *‘What others do’* regards that the barrier for PA is that a new generation is more passive, more striving for comfort and being at home. In addition, adolescents noted that among their surroundings there are very few who do PA every day.
4. **Factors outside** included the following subthemes:
  - *‘School duties first’* regards that PA barriers are in the priority that school sets in grades and in the need to fulfill school learning duties in the first place (i.e. lack of time and energy for PA), as well as fatigue from school.
  - *‘We are more online’* regards that PA enablers are sports motivational videos, sports groups, online posts, and mobile applications for doing physical exercises. As barriers adolescents pointed to the constant presence in the online world (e.g. Instagram, FB, Discord) and the inability to get out of it, as well as fatigue from mobile phones.
  - *‘We have a place for PA’* regards the importance of a PA-promoting environment that encourages adolescents to be physically active (e.g. distance to a sports club, availability of sports clubs, more opportunities for training in the city). As PA barriers it were identified insufficient funding for sports, the lack of sufficient sports grounds and the lack of space for sports at home.
  - *‘Inspire for PA’* regards that PA enabler is a sports role model in the surroundings and famous persons (e.g. fitness on Instagram, influencers, achievements of athletes). In addition, sports promotions were also noted as the enablers for PA.

## Discussion

The aim of this study was to explore the barriers and enablers for sufficient MVPA in adolescents from their point of view. Based on the statements of adolescents, four main themes were identified regarding factors contributing to (not) engagement in PA among adolescents. Adolescents noted the importance of their own efforts and physical predispositions, as well as knowledge and experience gained in the field of PA (‘Myself as a source’). The school is perceived by adolescents as a place offering an opportunity to be physically active, and PA teachers and sports coaches are perceived as specialists who can create favourable conditions for sports (‘How PA can be done’). A social circle—family, friends, and surroundings—who set a positive sports example and encourages adolescent’s efforts in PA (‘Others as a source’). External factors that can inspire adolescents to be physically active, give them a comfortable space and time to exercise, or be a barrier that minimizes this opportunity (‘Factors outside’).

### "I am the driving force of my actions"

Regarding the theme ‘Myself as a source’, our finding that adolescents perceive themselves as the main driving force for continuing or increasing the engagement in PA aligns with the self-determination theory, according to which if a sense of competence, autonomy, and relatedness are satisfied, individuals become more self-determined toward a given behavior [29]. In addition, this indicates that adolescents are not passive objects in PA, but rather show interest themselves, and have ideas and knowledge that directly affect the degree of their engagement in PA. Not the last role in this play their awareness about the health benefits of PA, weight management, and preserving a particular physical appearance, which is consistent with findings of Abdelghaffar et al. [12]. In addition, a lack of success in PA was found as an important facilitator/barrier for adolescents, potentially due to the competitiveness in adolescence. As noted by Swain and Jones [30], individuals who are strive to compete and achieve challenging sport-related goals are more likely to be engage in sports. Moreover, according to Keresztes et al. [31], adolescents who prefer to avoid social conflict and have a fear of competition may be an important target group for PA promotion programs. Furthermore, Vanhelst et al. [32] conclude that raising awareness among adolescents about the optional technique and intensity (“step-by-step”) of PA may promote behavior change in adolescents. It may also help to prevent PA barriers such as injuries and fear to be injured during PA by improving physical and health literacy among adolescents.

### “What is going on with our PA at school?”

Regarding the theme ‘How PA can be done at school’, we found that adolescents emphasized the role of the school as opportunity for PA as well as the importance of attitudes and expertise of teachers. According to WHO recommendations [33], adolescents should do at least an average of 60min/day of MVPA, however, adolescents constitute one of the most sedentary group of the population [34]. Here, a core issue is that adolescents spend most of the day at school, and their engagement in PA directly in the school space looks like a logical solution to this issue. Furthermore, we found that adolescents emphasized that the physical education should be more diverse, fun and friendly. This aligns with the findings of Baheiraei et al. [35] that adolescents became demotivated when they developed bad relationships with teachers and when teachers did not pay enough attention to PA. This indicates that adolescents lack positive emotions from PA as a part of their schooling, and they face such difficulties as overly competitive environment, lack of choice, providing activities that are not enjoyable and cannot be transferred to another context.

### “What is going on with our PA at sport clubs?”

The importance of organized sports activities under the supervision of qualified adults in achieving a sufficient level of PA and development of physical literacy should not be neglected. According to Hansen et al. [36], through structure, regular schedules, a focus on skill development, and adult supervision, adolescents experience higher levels of intrinsic motivation to be physically active. Additionally, we found that adolescents want a more playful way of PA with more games and diverse exercises in sport clubs. It is important to note, that according to adolescents, trainers’ personality can act as an enabler and a barrier for PA at the same time. This is consistent with the findings of Humbert et al. [20], in which were found that encouragement, feedback, and trainers’ overall knowledge work as enablers for PA and unfair trainers’ behavior is PA barrier for adolescents. In other words, adolescents expect adults to create safety and support for every student during PA, i.e. they need a safe place with enthusiastic PA teacher/trainer, where they can spend their time with joy without fear of rejection and punishment.

### “Who is my support and role model in PA?”

Regarding the theme ‘Others as a source’, we found that for adolescents it is crucial to get support from family and friends to start and maintain PA. Moreover, we found that building relationships with peers during sports and have mutual support within a team can contribute to staying engaged in PA. This aligns with previous research by Garcia et al. [37], which showed that adolescents who were more physically active had friends with significantly higher MVPA levels. According to Ketteridge and Boshoff [38], adolescents exercised in order to socialize, and exercise helped them to build relationships and to meet new people. Reversely, we found that unsuccessful experience in team sports could discourage adolescents to play sports.

On top, similar to Martins et al. [39] we found that an introduction to sports at an early age could help overcome the initial reluctance and fear to participate in PA, and similarly parents’ decision to involve their child in sport. Reversely, Fernández-Prieto et al. [40] noted that a sedentary lifestyle of parents has a strong demotivating effect and keeps adolescents away from engagement in PA. Finally, adolescents mentioned a generational change in which the new generation is more passive, more striving for comfort and being at home. Regarding this, their statement that in their surroundings very few are physically active every day indicates that adolescents still focus on how others behave. Abdelghaffar et al. [12] highlighted that lack of PA awareness in family, a lack of friends, having friends who are not physically active, and having friends with other interests may deter adolescents from PA. In other words, the descriptive norms might play a significant role as well.

### “What blocks and inspires me to be physically active?”

Regarding the theme ‘Factors outside’, we found that school duties, PA-promoting environment and the online world regard factors that can lead to well-developed and active lives for adolescents but can also block them from PA. First, our finding that adolescents had a fatigue from school and did not have time for PA because of their school duties is consistent with the findings of Rajaraman et al. [41], who identified academic pressure as a common barrier to PA. This may reflect that adolescents regard academic achievement as their priority because it is directly related to their future and career. As a result, adolescents neglected PA and did not have or take enough time regularly perform PA due to the time required for studying. However, previous studies revealed that PA was affirmatively correlated to better academic performance and can be used as an effective coping strategy [42, 43]. This demonstrates that we have an important target group of adolescents who need a help to find a better way to balance sports and academic achievement by controlling the pressure from the teachers and parents.

Second, our finding that adolescents need a PA-promoting environment that encourages them to be physically active, i.e. availability of sport and play facilities which are accessible by foot or public transport, is in line with previous research by Van Hecke et al. [44]. Moreover, adolescents noted a need to improve the condition and equipment of sports halls. Accordingly, Madarasova Geckova et al. [45] noted that 42% of Slovak schools reported the condition of school playgrounds to be poor. This may explain why adolescents reported many negative experiences during physical education, and, as a result, why they do not find it attractive. In other words, school administrators, coaches and teachers should allocate sufficient funds to sports in order to implement appropriate PA programs.

Third, adolescents highlighted that they are constantly in the online world and that for them it is hard to get out of that. However, Abdelghaffar et al. [12] reported that adolescents viewed the media as one of the main awareness-raising sources of PA. It means that social networks and online technologies may be a strong driving force in terms of encourage sport attractiveness among adolescents. In our study, adolescents noted such PA enablers as a fitness on Instagram, sports promotions, motivational videos and sports groups. This demonstrates that it is a natural environment for adolescents that affects them not only in the online world, but also may have a significant impact on their offline world. Evidently, the involving media for raising PA awareness among adolescents requires further study regarding its underlying mechanisms.

#### Strengths

The main strength of this qualitative study is the examining the factors contributing to (not) engagement in PA using the viewpoints and experiences of adolescents in Slovakia. Another strength is that the collaborative work of the research team on code analysis and creating the final model of the barriers and enablers for sufficient MVPA in adolescents helped to avoid the subjectivity of the results obtained. A further strength is that we used semi-structured interviews that provided the opportunity to create an interactive conversation, helped participants to verbalise their responses and enabled reflecting on the consistency and diversity of statements. Finally, we used consensual qualitative research (CQR) methods, which have been shown to be an effective and valid method in gathering qualitative data among adolescents [24].

#### Limitations

Some limitations should also be mentioned. First, in our sample, we had few boys and less physically active adolescents, which could imply an insufficient heterogeneity of the sample. This was due to the fact that our qualitative studies were conducted during the second wave of the Covid-19 pandemic in Slovakia. During that wave government measures did not allow us to convene with participants personally. However, we reached saturation of the topic under study. This suggests that the potential bias because of this was limited as far as groups were included, but the above may affect the generalisability of our findings, in particular regarding groups that were underrepresented. Another limitation was that the analysis and interpretation of the data may have been affected by the researchers’ subjectivity. However, this potential bias was reduced by reaching consensus among several researchers on the identified topics and interpretations. Nevertheless, further research could have yielded more detailed information on factors contributing to (not) engagement in PA in adolescence.

#### Implications

Our findings have several implications. First, our finding that adolescents identified themselves as the main driving force for engagement in PA. Intervention might build on it and should aim at developing a physical literacy, offering safe opportunities to try various sports, enabling experiencing and reflecting feelings associated with being active, gathering knowledge needed to build and maintain physical competence.

Second, our finding that adolescents are highly aware of individual differences and physical predispositions implies that in order to attract and support active lifestyles among adolescents, coaches and PA teachers should place greater emphasis on ensuring that interventions are tailored to adolescents. They should further be attentive to the specific needs of subgroups with different physical predispositions and physical skills.

Third, based on our findings, the social environment plays a crucial role in the (not) engagement of adolescents in PA. Adolescents expect adults not just to be a role model, but also to create a safe, inclusive, supportive, and enjoyable environment for engaging in PA, and to be able to eliminate unwanted interpersonal behaviours of peers or adults in PA environment. This implies that interventions considering the impact of social circle in the engagement in PA might be more successful, i.e. parents need to be involved in PA programs to increase their impact on their children’s PA levels.

Fourth, our finding that the online world is a natural environment for nowadays adolescents implies that PA professionals can use the capacity of new technologies in terms of various devices, applications or online space to better fit into the environment in which adolescents live their lives. In other words, the use of social networks, motivational videos and PA apps can work as “a bridge” to promote engagement of youth in PA.

Finally, our qualitative approach definitely adds to our understanding of adolescents (not) engagement in PA. Thus, mixed method studies may help to combine the advantages of both approaches, quantitative as well as qualitative.

## Conclusion

We identified four themes regarding factors affecting engagement in PA in the perspectives of adolescents themselves. ‘Myself as a source’ represents the importance of adolescents’ own efforts and physical predispositions, as well as knowledge and experience gained in the field of PA. ‘How PA is done’ represents school as a place where there is an opportunity to be physically active, and PA teachers and sports coaches who are perceived as specialists who can create favourable conditions for sports. ‘Others as a source’ represents the social circle–family, friends, and surroundings–who set a positive sports example and encourages adolescent’s efforts in PA. ‘Factors outside’ represents external factors that can inspire adolescents to be physically active, give them a comfortable space and time to exercise, or be a barrier that minimises this opportunity. The potential factors that include adolescents’ perspectives can be more leveraged in designing supportive, inclusive, enjoyable, and skills-appropriate PA programmes.

## Supporting information

S1 Table Factors contributing to (not) engagement in PA among adolescents- in English language. (DOCX)

S2 Table Factors contributing to (not) engagement in PA among adolescents- in Slovak language. (DOCX)
