# Supplementary material for: Barriers and enablers for sufficient moderate-to-vigorous physical activity: The perspective of adolescents
Source: PLoS One. 2024 Feb 16;19(2):e0296736. doi: 10.1371/journal.pone.0296736 (PMC10871508; doi:10.1371/journal.pone.0296736)
Supplement: S1 Table — (DOCX) [file pone.0296736.s001.docx]

| **S1 Table. Factors contributing to (not) engagement in PA among adolescents- in English language.** |
| --- |
| **RESOURCES IN US, PERSONALITY** |
| **Goal setting as a motivator** – (10) |
| **(In)ability to commit and endure** / Self-denial as a facilitator / barrier (to be able to move in gradual steps; to move on from the comfort zone; good feeling of overcoming; determination and perseverance; to go through the pain; less laziness and comfort; to go through "I do not want") – (25) |
| **Quickly losing interest in the activity** (trains for 5 minutes and no longer enjoys it) – (3) |
| **Laziness** – (10) |
| **Having a relationship with sports** / activity (always finds time to move; energetic; needs to drain energy; likes sports; sports type; wants to do sports) – (5) |
| **Has other hobbies** (painting, music, computer games, learning, walking outside, social networks, watching a movie, reading) – (9) |
| **Mindset** – (4) |
| **BODY DECIDES** |
| **Does PA, but he doesn't have the physical condition** – (2) |
| **Fitness loss after a break in PA as a barrier** – (1) |
| **Overweight can motivate but also demotivate to PA** – (2) |
| **Injuries, fear of injuries discourage PA or prevent PA** – (5) |
| **Health problems as a barrier to PA** – (6) |
| **Predisposition,** **skills, fitness condition helps** (talent; genes; you can also be fit, but you do not know how to train; not everyone has the prerequisites to be a runner; they do not know how to catch balls; they are built for sports) – (15) |
| **I NEED TO KNOW HOW TO DO IT** |
| **They do not know how much and how to exercise** (they go from bed to computer, do three exercises for abs and done; they count the walk as PA; they do one exercise around and it does not help; they do not exercise as much as they think; they do those exercises badly; they cannot do time breaks correctly; they go running once per month; not enough activity) – (15) |
| **Find time for sport, planning** – (10) |
| **Music helps** – (2) |
| **Choose a suitable load and frequency** (not so strict and difficult; do not do training 5 times per week, because they will lose their own time, overtime training) – (5) |
| **Regular rest for regeneration** – (1) |
| **Master the technique and get fit gradually** – (4) |
| **Regularity and perseverance are required to remain at PA** – (2) |
| **Diet and sleep affect PA** – (4) |
| **Movement as a habit** – (5) |
| **Be able to try different physical activities** / Allow to try sports – (15) |
| **Find a suitable sport he likes** (find a suitable activity, sports advice) – (14) |
| **Understanding and finding meaning of PA** – (5) |
| **HOW DO WE EXPERIENCE IT** |
| **Success motivates** / Feeling of success (when I am good at it; has talent; they want to win; want to be the best; achievements) – (12) |
| **Progress as motivation** – (5) |
| **Failure demotivates** (they are not bad, they do not see the results; he fails; he does not know how to catch balls; he does not know how to do the sport; they have no talent and they are suffering) – (14) |
| **I like sports even though I do not succeed** – (1) |
| **Lack of experience** (what it is like to improve; what it is like to catch up) – (2) |
| **Good feeling after exercise** – (2) |
| **Someone supports and motivates me** – (8) |
| **PA AFFECTS HEALTH** |
| **Positive impact of sports on the mental health motivates** (forget about their problems; endorphins; relieves stress; relax; turn off; clear your head; it is good for the brain; distraction from depression; drain energy) – (6) |
| **Positive impact of sports on the physical health motivates** (compensation of sitting; blood circulation; vitamins; if PA is outside - vitamin D; spine; better sleep) – (11) |
| **Health and body as a motive** (I want to look better; feel good in my body; be fit; have muscles; have a good figure; be healthy; I want to lose weight) – (15) |
| **WHAT DOES OTHERS DO? (Descriptive standards)** |
| **Generational change** (new generation is more passive, looking for more comfort) – (7) |
| **I know very few people who do PA every day** – (5) |
| **Everyone is at home** – (4) |
| **MY FRIENDS** |
| **I am going where my friends are** – (10) |
| **I do not like to do it (PA) alone** – (5) |
| **Invite a friend to play sports** – (2) |
| **Friend's support** (friend supports me; I can talk to him about it; encourages me) – (11) |
| **Creating relationships with peers from training** (go to pizza after training; get to know each other; sports community) – (8) |
| **Team support in team sports** – (4) |
| **The team influence** (“I have friends there”) – (9) |
| **Bad experience discourages from sports** (they do not feel good in that team; they make fun of you; they are mean to you) – (7) |
| **MY FAMILY** |
| **Parents or family as a role model** – (6) |
| **Parents' decision initiates PA and helps to overcome initial obstacles** – (9) |
| **Parents’ pressure demotivates to do PA** – (4) |
| **My parents support me in PA** – (8) |
| **Parents teach their children to play sports from an early age** – (15) |
| **Family does not have money for sports clubs** (family economic situation) – (2) |
| **If someone does not play sports as a child, it will be difficult in older age** – (15) |
| **PA AT SCHOOL** |
| **More hours of PA in schools** / Physical education is not enough in schools – (2) |
| **PA in schools as a duty and boredom, not joy** (all year play only football; cancel marks; devote individually and in smaller groups; I love sports, but not in school; they are suffering with activities that don't suit them) – (7) |
| **PA in schools needs change** (in smaller groups; more fun; no competitions; to choose from more sports; to get acquainted with sports; more friendly PA; to skip marks; to adapt to the physical possibilities of students) – (9) |
| **Teacher as a facilitator** (to know about students' talent; teacher motivates to PA; offer activity; younger teachers have more enthusiasm; teacher's approach) – (3) |
| **School as a place for sports** (more spots clubs; possibility to play ping pong or darts during breaks; place for interventions) – (3) |
| **Condition and equipment for gyms in schools** – (3) |
| **PA AT OTHER OCCASIONS** |
| **Personality, experience and attitude of the coach** (to give an individual feedback; adapt the training to physical possibilities so that everyone can handle it; to build a friendly relationship; leave due to inappropriate behaviour of the coach; the coach has experience; the fair coach; does not make too much pressure; has authority but stay friendly) – (16) |
| **When PA is fun** / To make PA fun (fun, playful, entertaining; in a form of bitingly interesting; to invent something new all the time; to keep them entertained) – (10) |
| **The attractiveness of the sport** (know somersaults and tricks; it catches the eye; trends) – (7) |
| **Some sports are expensive** – (4) |
| **WE HAVE A PLACE FOR PA** |
| **Sports centers** (better training there, popularized on Instagram) – (2) |
| **Available sports clubs** – (4) |
| **Missing space to exercise at home** – (1) |
| **Lack of sports grounds** (availability of playground) – (2) |
| **There are more opportunities in the city** – (3) |
| **PA-promoting environment** – (3) |
| **Insufficient sports funding** – (2) |
| **SCHOOL OBLIGATIONS, MARKS, FATIGUE** |
| **Fatigue from school** – (2) |
| **School grades more important than PA** – (1) |
| **School duties first** – (11) |
| **WE ARE MORE ONLINE** |
| **Everyone is online** – (3) |
| **Fatigue from mobile** – (1) |
| **Videos, groups and posts on the internet give motivation** – (10) |
| **“Falling” into the online network and inability to escape from it** – (5) |
| **Online learning gives more time to make PA** – (1) |
| **PA Apps** – (1) |
| **INSPIRATE FOR PA** |
| **Sports campaigns as a support** (campaigns; raising awareness; making it necessary to play sports) – (4) |
| **Sports model in someone around** (take an example from someone; stories and photos on the Instagram; tell your story; coach as a role model; coach will invite athletes who are PA role models) – (7) |
| **Famous person as a role model** (fitness on Instagram; influencers; Attil Vegh, Sagan, Vlhova; achievements of athletes) – (9) |
