# Supplementary material for: Barriers and enablers for sufficient moderate-to-vigorous physical activity: The perspective of adolescents
Source: PLoS One. 2024 Feb 16;19(2):e0296736. doi: 10.1371/journal.pone.0296736 (PMC10871508; doi:10.1371/journal.pone.0296736)
Supplement: S2 Table — (DOCX) [file pone.0296736.s002.docx]

| **S2 Table. Factors contributing to (not) engagement in PA among adolescents- in Slovak language.** |
| --- |
| **ZDROJE V NÁS, OSOBNOSŤ** |
| **Stanovenie cieľa ako motivátor –** (10) |
| **(Ne)schopnosť odhodlať sa a vydržať** /Sebazaprenie ako facilitátor/bariéra (vedieť sa posúvať v postupných krokoch, posúvať komfortnú zónu, dobrý pocit z prekonania sa, odhodlanosť a vytrvalosť, ísť cez bolesť, menej lenivosti a pohodlnosti, ísť za „sa mi nechce“, za bolesť, dokopať sa – (25) |
| **Rýchlo stratí záujem o aktivitu** (5 minút cvičí a už ho to nebaví) – (3) |
| **Lenivosť** – (10) |
| **Mať vzťah k športu, aktívnosť** (vždy si nájde čas na pohyb, energický, potrebuje vybiť energiu, má rád šport, športový typ, chce robiť šport) – (5) |
| **Má iné koníčky** (iné hobby, maľovanie, hudba, počítačové hry, učenie, chodenie vonku, sociálne siete, pozrie film, čítanie) – (9) |
| **Mindset nastavenie cloveka** – (4) |
| **ROZHODUJE TELO** |
| **Cvičí** a**le nemá kondíciu –** (2) |
| **Strata kondície po prestávke v PA ako bariéra –** (1) |
| **Nadhmotnosť môže motivovať ale aj demotivovať k PA** – (2) |
| **Úrazy, obavy z úrazov** **odrádzajú od PA, alebo znemožňujú PA** – (5) |
| **Zdravotné problémy ako bariéra PA** – (6) |
| **Predispozícia, zručnosť, kondícia pomáha** (talent, gény, kotrmelce, šplhanie, môžeš mať aj kondíciu ale šplhať nevieš, nie každý má predpoklady byť bežec, nevie chytať lopty, je stavaný na športy – (15) |
| **POTREBUJEM VEDIEŤ AKO NA TO** |
| **Nevedia koľko a ako cvičiť** (prejdú od postele k počítaču, spravia tri brušáky, prechádzku počítajú ako PA, robia jeden cvik dookola a to nepomáha, necvičia tak veľa ako si myslia, robia tie cviky zle, nevedia si načasovať prestávky správne, idú behať raz za mesiac, aktivita nie je dostačujúca – (15) |
| **Vedieť si** **nájsť si čas na šport, plánovanie –** (10) |
| **Hudba pomáha –** (2) |
| **Zvoliť primeranú záťaž a frekvenciu** (nie tak prísne a ťažké, nie 5x do týždňa tréning lebo prídu o vlastný čas) – (5) |
| **Pravidelný oddych na regeneráciu –** (1) |
| **Osvojiť si techniku a získať kondíciu postupne –** (4) |
| **Na zotrvaní pri PA je potrebná pravidelnosť a vytrvalosť –** (2) |
| **Strava a spánok vplýva na PA –** (4) |
| **Pohyb ako návyk –** (5) |
| **Môcť si vyskúšať rôzne fyzické aktivity**/ Umožniť vyskúšať šport – (15) |
| **Nájsť si/mu vhodný šport, ktorý sa mu páči** (nájsť vhodnú aktivitu, športové poradenstvo) – (14) |
| **Porozumenie a nájdenie zmyslu PA** – (5) |
| **AKO TO PREŽÍVAME** |
| **Úspech motivuje**/ Pocit úspechu (keď mi to ide, som v tom dobrý, má talent, chcú vyhrať, byť najlepší, úspechy) – (12) |
| **Progres ako motivácia** – (5) |
| **Neúspech, nezručnosť demotivuje** (sú nemehlá, nevidieť výsledky, nedarí sa mu, nevie chytať lopty, nevie robiť ten šport, nemajú nadanie a morduje sa – (14) |
| **Mám rád šport aj keď sa mi to nedarí –** (1) |
| **Chýba skúsenosť s prežívaním** (aké to je zlepšovať sa, aké to je dobehnúť) – (2) |
| **Dobrý pocit po cvičení –** (2) |
| **Niekto ma podporuje, motivuje, teší sa zo mňa** – (8) |

| **VPLYV PA NA ZDRAVIE MOTIVUJE** |
| --- |
| **Pozitívny vplyv športu na duševné zdravie motivuje** (zabudnú na svoje problémy, endorfíny, zmierňuje stres, odreagovanie, vypnem, vyčistiť hlavu, pre mozog je to dobré, rozptýlenie od depresie, vybiť energiu) – (6) |
| **Pozitívny vplyv športu na fyzické zdravie motivuje** (kompenzácia sedenia, krvný obeh, vitamíny, je na slnku tak D vitamin, chrbtica, lepší spánok) – (11) |
| **Zdravie a telo ako motív** (chcem lepšie vyzerať, dobre sa cítiť v svojom tele, mať kondíciu, mať svaly, mať dobrú postavu, byť zdravý, chcem schudnúť) – (15) |
| **ČO ROBIA INÍ? (DESKRIPTÍVNE NORMY)** |
| **Generačná zmena** (nová generácia je pasívnejšia, pohodlnejšia) – (7) |
| **Poznám veľmi málo ľudí, ktorí robia PA každý deň** – (5) |
| **Všetci sú doma** – (4) |
| **MOJI KAMARÁTI** |
| **Idem tam, kde sú kamoši** – (10) |
| idem von s kamaratmi – (4) |
| **Samého ma to nebaví –** (5) |
| **Prizvať kamaráta k športu** – (2) |
| **Podpora kamaráta** (kamarát ma podporuje, teší sa, môžem sa s ním o tom porozprávať, povzbudí ma) – (11) |
| **Vytvorenie vzťahov s rovesníkmi na tréningoch** (po tréningu ísť na pizzu, porozprávať sa, spoznávať sa, športová komunita) – (8) |
| **Tím, vzájomná podpora v tímových športoch –** (4) |
| **Vplyv kolektívu** (mám tam kamarátov, ten kolektív ...) – (9) |
| **Zlý zážitok odradí od športu** (necíti sa dobre v tom kolektíve, nevyberajú si ho do tímu, robia si z vás srandu, sú na neho zlí) – (7) |
| **MOJA RODINA** |
| **Rodičia, alebo rodina ako role model** – (6) |
| **Rozhodnutie rodičov iniciuje PA a pomáha prekonať počiatočné prekážky** – (9) |
| **Tlak rodiča znechutí PA** – (4) |
| **Rodičia ma podporujú v PA –** (8) |
| **Rodičia vedú k športu od malička –** (15) |
| **Rodina nemá peniaze na krúžky** (ekonomická situácia v rodine) – (2) |
| **Ak niekto nešportuje v detstve, tak ťažko bude neskôr –** (15) |
| **PA NA ŠKOLÁCH** |
| **Viac hodín TV na školách**/ Telesná výchova na školách málo – (2) |
| **TV na školách ako povinnosť a nuda, nie radosť** (venovať sa indiviudálne a v menších skupinkách, prispôsobiť sa čomu fyzicky stačia, nie trest a a nuda, z donútenia, šport milujem ale TV v škole ma nebaví, mordujú sa s aktivitami, ktoré im nejdú) – (7) |
| **TV na školách potrebuje zmenu** (v menších skupinách, zábavnejšie, súťaže, dať na výber z viacerých športov, zoznámiť so športami, priateľskejšie, zrušiť známkovanie, prístup učiteľov, prispôsobiť fyzickým možnostiam žiakov) – (9) |
| **Učiteľ ako facilitátor** (vedieť o talente žiakov, učiteľ motivuje k PA, ponúknuť aktivitu, mladší s entuziazmom) – (3) |
| **Škola ako miesto pre šport** (viac krúžkov, možnosť hrať pingpong alebo šípky cez prestávky, miesto pre intervencie) – (3) |
| **Stav a vybavenie telocviční v školách –** (3) |

| **PA MIMO ŠKOLY** |
| --- |
| **Osobnosť, skúsenosti a prístup trénera** (mať trénera, dáva individuálnu spätnú väzbu, prispôsobiť tréning fyzickým možnostiam aby to každý zvládol, vytvorí si priateľský vzťah, odísť kvôli nevhodnému správaniu trénera, tréner má skúsenosti, je spravodlivý, neznevažujú, prílišný tlak, krik, tresty, mať autoritu ale ostať priateľský, podporuje vs. deptá, „čo ty tu chceš?) – (16) |
| **Keď je PA zábavná/** Aby bola PA zábavná (sranda, hravá, zábavná, formou hierzaujímavá, vymýšľať stále nové, radosť, aby ich to bavilo – (10) |
| **Aktraktivita športu** (že vidia saltá a triky, upúta, je to v móde) – (7) |
| **Niektoré športy sú drahé** – (4) |
| **MÁME KDE ŠPORTOVAŤ (PODMIENKY NA PA)** |
| **Športové centrá** (lepšie sa tam trénuje, popularizované na instagrame) – (2) |
| **Dostupné športové krúžky –** (4) |
| **Chýba priestor na cvičenie** **doma –** (1) |
| **Nedostatok športovísk** – (2) |
| **V meste je viac príležitostí** – (3) |
| **Prostredie vyzývajúce k PA** – (3) |
| **Nedotatočné financovanie športu** – (2) |
| **ŠKOLSKÉ POVINNOSTI, ÚNAVA, ZNÁMKY NA ÚKOR PA** |
| **Únava zo školy –** (2) |
| **Školské známky dôležitejšie než PA** – (1) |
| **Školské povinnosti na úkor PA** – (11) |
| **SME VIAC ONLINE** |
| **Všetci sú online** – (3) |
| **Únava z mobilov –** (1) |
| **Videa, skupiny a posty na nete motivujú** – (10) |
| **Spadnutie do online siete a neschopnosť sa vymaniť –** (5) |
| **Dištančné vzdelávanie (viac času pre pohyb) –** (1) |
| **Aplikácie na PA –** (1) |
| **INŠPIROVAŤ K PA** |
| **Športové kampane ako podporný prostriedok** (kampane, osveta, dať najavo že treba športovať) – (4) |
| **Športový vzor v niekom z okolia** (brať si príklad z niekoho, príbehy a fotky na instagrame, povedať svoj príbeh, tréner ako role model, tréner pozve športovcov, kt sú role model) – (7) |
| **Známa osobnosť ako role model** (fitnesska na instagrame, Attil Vegh, influenceri, Sagan, Vlhova, úspechy športovcov) – (9) |
